# Shared decision making between older people with multimorbidity and GPs: a qualitative study

**DOI:** 10.3399/BJGP.2021.0529

**Published:** 2022-04-05

**Authors:** Emily L Brown, Leon Poltawski, Emma Pitchforth, Suzanne H Richards, John L Campbell, Joanne E Butterworth

**Affiliations:** National Institute for Health Research doctoral research fellow and GP, Exeter Collaboration for Academic Primary Care, College of Medicine and Health, University of Exeter, Exeter.; National Institute for Health Research doctoral research fellow and GP, Exeter Collaboration for Academic Primary Care, College of Medicine and Health, University of Exeter, Exeter.; National Institute for Health Research doctoral research fellow and GP, Exeter Collaboration for Academic Primary Care, College of Medicine and Health, University of Exeter, Exeter.; Division of Primary Care, Palliative Care and Public Health, Faculty of Medicine and Health, University of Leeds, Leeds Institute of Health Sciences, Leeds.; National Institute for Health Research doctoral research fellow and GP, Exeter Collaboration for Academic Primary Care, College of Medicine and Health, University of Exeter, Exeter.; National Institute for Health Research doctoral research fellow and GP, Exeter Collaboration for Academic Primary Care, College of Medicine and Health, University of Exeter, Exeter.

**Keywords:** general practice, multimorbidity, aged, shared decision making, qualitative research

## Abstract

**Background:**

Shared decision making (SDM), utilising the expertise of both patient and clinician, is a key feature of good-quality patient care. Multimorbidity can complicate SDM, yet few studies have explored this dynamic for older patients with multimorbidity in general practice.

**Aim:**

To explore factors influencing SDM from the perspectives of older patients with multimorbidity and GPs, to inform improvements in personalised care.

**Design and setting:**

Qualitative study. General practices (rural and urban) in Devon, England.

**Method:**

Four focus groups: two with patients (aged ≥65 years with multimorbidity) and two with GPs. Data were coded inductively by applying thematic analysis.

**Results:**

Patient acknowledgement of clinician medicolegal vulnerability in the context of multimorbidity, and their recognition of this as a barrier to SDM, is a new finding. Medicolegal vulnerability was a unifying theme for other reported barriers to SDM. These included expectations for GPs to follow clinical guidelines, challenges encountered in applying guidelines and in communicating clinical uncertainty, and limited clinician self-efficacy for SDM. Increasing consultation duration and improving continuity were viewed as facilitators.

**Conclusion:**

Clinician perceptions of medicolegal vulnerability are recognised by both patients and GPs as a barrier to SDM and should be addressed to optimise delivery of personalised care. Greater awareness of multimorbidity guidelines is needed. Educating clinicians in the communication of uncertainty should be a core component of SDM training. The incorrect perception that most clinicians already effectively facilitate SDM should be addressed to improve the uptake of personalised care interventions.

## INTRODUCTION

The population is ageing and consequently the ‘older’ age group (aged ≥65 years) is widening. The prevalence of multimorbidity (≥2 long-term conditions) in older people is high and predicted to rise.^1^^,^^2^ Older patients with multimorbidity have higher rates of disability and functional decline, increased mortality, and reduced wellbeing when compared with younger, healthier patients.^3^^–^7

Clinical decision making with older patients with multimorbidity can be complex and challenging.^8^^–^12 Older patients with multimorbidity have high primary care usage and increased costs of care when compared with younger, less complex patients.^13^^,^^14^ Providing care to this patient group contributes significantly to time and workload pressures experienced by GPs.^15^

Older patients value a trusting relationship with their GP, respecting the GP’s expertise in the context of clinical decision making.^16^ However, they also appreciate involvement in decision making about their care.^17^^,^^18^ Patient-reported barriers to such involvement include perceived power imbalances in the doctor– patient relationship,^19^ poor practitioner communication skills,^20^ and patients’ perceptions that primary care clinicians do not recognise the patient’s expertise in their own health.^21^ Successful shared decision making (SDM) centres around the respective expertise of the patient and the healthcare professional and relies on effective engagement by both parties.^22^

SDM is recognised as a core component of personalised, patient-centred care, both nationally^23^ and globally,^24^^–^27 and is advocated in clinical guidelines for the management of multimorbidity.^28^ The NHS Long Term Plan aims for personalised care for 2.5 million people by 2024.^29^^–^31 SDM has benefits in terms of improving patient’s trust in their doctor, their satisfaction with health care, and their adherence to treatment advice.^32^^–^36 However, it is not yet commonplace, with estimates that SDM is only used in 10% of applicable situations.^37^ Clinicians commonly, yet incorrectly, perceive that effective SDM has been achieved.^38^

Few studies evaluate the effectiveness of interventions that facilitate SDM for older patients with multimorbidity during general practice consultations.^39^ Recent guidance from the National Institute for Health and Care Excellence (NICE) recommends research to explore what influences the acceptability of patient involvement in decision making in populations that predominantly believe in the authority of healthcare professionals.^40^ Therefore, this study used qualitative methods to explore the perceptions and experiences^41^ of older patients with multimorbidity and GPs when seeking to achieve this core component of personalised care.

**Table:** How this fits in

Few studies have explored potential barriers to shared decision making (SDM) from the perspective of both older patients with multimorbidity and GPs. Patient acknowledgement of clinician medicolegal vulnerability in the context of multimorbidity, and recognition of this as a barrier to SDM, is a new finding. Medicolegal vulnerability was a unifying theme for other barriers commonly reported to be influencing consulting behaviours by both patients and GPs. GPs need support and training in communicating clinical uncertainty and in utilising multimorbidity guidelines in order to deliver effective, personalised care.

## METHOD

The study was undertaken in the context of refining a new intervention (VOLITION)^42^ to facilitate the involvement of older patients with multimorbidity in decision making during GP consultations. VOLITION consisted of two draft components: a patient leaflet, to facilitate patients to convey their preferences for involvement to the GP; and a GP workshop, training GPs in SDM communication skills.

Ethical approval was obtained from the Health Research Authority. Patient and public involvement (PPI) was sought during study design and when refining patient-facing documents. The consolidated criteria for reporting qualitative research (COREQ) was applied.^43^ Participants were sampled from four general practices, rural and urban, in Devon, England. GPs were approached by email via the local Clinical Research Network, provided with an information sheet, and screened for eligibility (Table 1). Practices were offered payment for GPs’ time and for administrative procedures.

**Table 1.** Participant inclusion and exclusion criteria

**Inclusion criteria**	**Exclusion criteria**
**Patients**	
≥2 long-term health problems^a^	Temporary residents
	Vulnerability from a recent bereavement
	Severe mental illness
	Reduced cognitive ability
	Extreme frailty or end stage disease
	Severe communication impairment
	Learning disability

**GPs**	
Permanent GPs from the recruited practices (including partners or salaried staff)	GP trainees
	Locum GPs
Any working hours	Junior doctors working in general practice

a *Conditions included were: angina or long-term heart problem; arthritis or long-term joint problem; asthma or long-term chest problem; blindness or severe visual impairment; cancer in the last 5 years; deafness or severe hearing impairment; diabetes; epilepsy; high blood pressure; kidney or liver disease; long-term back problem; long-term mental health problem; and long-term neurological problem. No minimum time for a long-term condition.*

Practice administrators identified patients aged ≥65 years with ≥2 long-term conditions using a computer algorithm. They purposively sampled patients to ensure variation by number of health conditions. Forty patients per practice were contacted by post and requested to respond within 4 weeks. Patients were offered travel expenses and refreshments during focus groups. GPs screened potential patient participants against exclusion criteria (Table 1).

Eligible participants were contacted by phone (patients) or email (GPs). Patient focus groups were held at the University of Exeter. GP focus groups were held within participating practices. Written, informed consent was obtained from all participants, with confidentiality guaranteed. Four focus groups were held (May 2019), with four participants per group, two groups with GPs and two with patients. Focus groups contained participants from multiple practices.

One author guided discussion using a topic guide (see Supplementary Box S1 and S2), while another author took fieldnotes. Participants had no previous knowledge of the former author, though participants knew the latter was a GP. Participants were asked to identify improvements to the proposed intervention (GPs considered a training workshop; patients reviewed a handout). Participants were also asked to discuss potential facilitators and barriers to patient involvement in decision making for older patients with multimorbidity. These latter findings, from both patient and GP perspectives, are the focus of this study.

Focus groups were audiorecorded, and transcribed externally under a confidentiality agreement. NVivo (version 12 plus) aided coding.^44^ Audiorecordings, transcription files, and fieldnotes provided an audit trail.

Thematic analysis was undertaken to rigorously identify patterns of meaning across the dataset, through coding of data, and the development and revision of common themes.^45^ Data were coded inductively. Categorising the data into interpretative themes was an iterative process undertaken during data coding. Dissonant views were specifically sought. Data from patient and GP focus groups were initially coded separately. However, common themes were identified across all four groups, leading to the generation of interpretative themes relevant to both patients and GPs. Two researchers coded data independently before comparing, ensuring consistency of coding. Both coders applied reflexivity in their interpretation of findings,^46^ considering how their experiences as clinicians influenced their interpretation of the data. While additional focus groups were not planned, the number of participants and length of focus groups allowed the topic to be well-covered, and on completion of coding the researchers agreed that no new themes were emerging and that saturation had been achieved.^44^ Member checking did not take place due to time and resource constraints. However, the PPI group considered the validity of patient-reported themes from a lay perspective, and the GP-academics on the research team considered GP-reported themes critically.

## RESULTS

**Figure 1.**
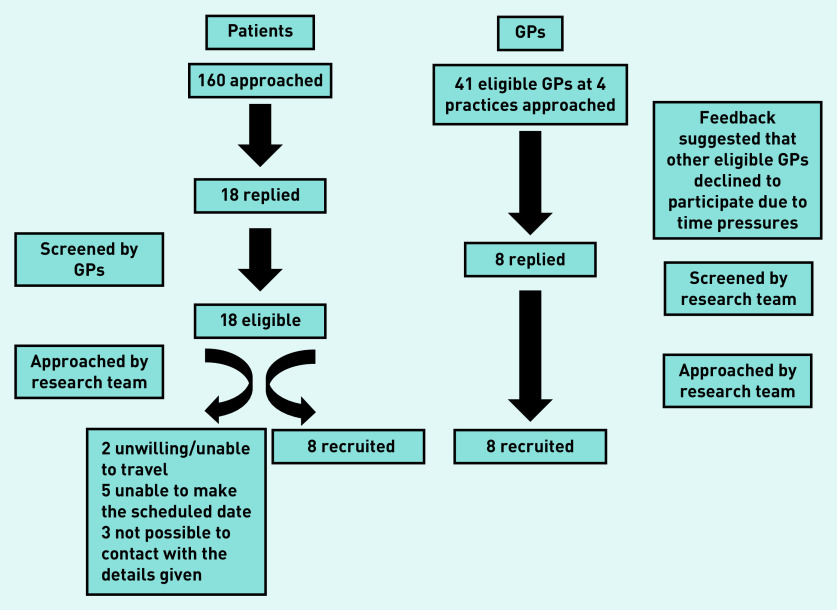
*Participant recruitment.*

**Figure 2.**
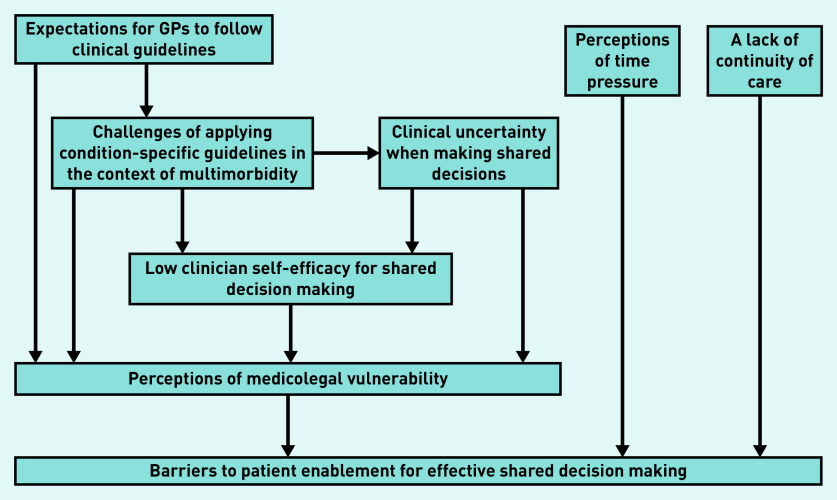
*Medicolegal vulnerability as a unifying theme for potential barriers to shared decision-making between older patients with multimorbidity and GPs.*

The flow of recruitment is presented in Figure 1. Characteristics of the 16 participants are presented in Table 2. Each group discussion lasted 1.5 hours. Common themes across participant groups are presented together and summarised in Figure 2.

**Table 2.** Participant characteristics

**Participants**	***n***
**Patients**	8

**Age, years**	
65–74	6
75–84	2

**Sex**	
Male	1
Female	7

**Ethnic group**	
White British	8

**Number of long-term health problems**	
2	3
3	4
≥4	1

**Location of general practice**	
Urban	5
Rural	3

**GPs**	8

**Time since qualification, years**	
<5	1
5–10	4
>10	3

**Sex**	
Male	1
Female	7

**Ethnic group**	
White British	7
Asian	1

**Location of general practice**	
Urban	6
Rural	2

Patients and GPs recognised that enabling patients to take part was central to the attainment of high-quality SDM. One GP felt that patients could be educated to expect involvement, wishing to advise patients that:

*‘Your doctors are there to help you: They will discuss with you, and if you decide what they’ve decided isn’t what you want, that’s fine as well. They are the experts on health, but you’re the expert in terms of what you want.’*

However, both participant groups reflected that, due to the factors outlined below, patient enablement was not always achieved. Patients voiced disappointment in this respect:

*‘I like to have lots of options in front of me so that I know I can make an informed decision. But I don’t feel like that way when I go to the doctor, I feel dis-empowered.’*

Both patients and GPs identified a strong common barrier to effective SDM, which, for the purposes of this study, is termed ‘medicolegal vulnerability’, and a number of subthemes are described. Patient and GP participants also reflected on the impact of time pressures and the relevance of continuity of care when discussing factors influencing SDM.

### Medicolegal vulnerability

The term ‘medicolegal vulnerability’ is used to reflect a doctor’s concerns about being open to professional or legal challenges in the event of negative outcomes from the doctor’s clinical management of a patient. Several subthemes were identified in relation to medicolegal vulnerability (Figure 2): expectations for GPs to follow clinical guidelines; challenges of applying condition-specific guidelines in the context of complex multimorbidity; managing clinical uncertainty when facilitating shared decisions; and clinician self-efficacy for SDM.

#### Expectations for GPs to follow clinical guidelines

GPs reported that the ‘constraints’ of following clinical guidelines could limit opportunities to apply an individualised, patient-centred approach to the decision-making process for patients whose personal priorities and individual characteristics did not always relate to the available evidence base. They stated that:

*‘If you step out of line … you haven’t got a leg to stand on, even though they’re only guidelines. So, actually … the decisions are being made for them.’*

Pay-for-performance criteria contributed to GPs’ feelings of being obligated to follow guidelines, despite uncertainty surrounding their applicability to the individual patient, as one GP recalled:

*‘QOF* [Quality and Outcomes Framework] *tells me this … but I have no idea because you’re* [aged] *90. So in theory it could be or in theory it could not be.’*

GPs voiced a desire to be permitted to use their knowledge of the patient to apply a personalised approach to care and to consider options holistically with the patient. However, GPs appeared unaware of guidelines supporting this, such as the NICE multimorbidity guidelines.^28^

Both patients and GPs perceived that adherence to guidelines could protect GPs from blame in the event of negative consequences from clinical decision making. GPs discussed their feelings that, *‘If we don’t follow* [guidelines] *, we lay ourselves open to being sued.’* Patients recognised that population-level recommendations did not always apply to their patient group. They appeared to value the opportunity for a more personalised approach to clinical decision making but did not expect it, as they voiced concerns that, *‘if something goes wrong, they* [the patient] *are twisted the other way, and the poor doctor gets the blame for it in the end.’*

GPs reported potential risks of over-treatment for patients with multimorbidity through strict adherence to multiple, condition-specific clinical guidelines:

*‘Isn’t it a matter that they’re* [patients] *getting over-treated, perhaps, because we will do what the guidelines suggest?’*

They recognised that conservative management can be a valid outcome of a shared decision when consulting with this patient group, particularly when seeking to reduce the possibility of doing harm:

*‘Yes, and we don’t give them the opportunity to say no always.’*

#### Challenges of applying condition-specific guidelines in the context of complex multimorbidity

Both GPs and patients recognised the importance of adequately discussing risk regarding mutual decisions to avoid medicolegal vulnerability associated with any negative outcomes. GPs discussed examples of difficulties encountered when considering whether they had *‘properly consented that person for the decision they’re making’,* saying *, ‘That’s where guidance comes in handy.’* Patients discussed hypothetical examples of where the GP *‘didn’t tell me this was likely to happen’* and their awareness of media coverage of such scenarios, stating, *‘You hear of that, you read it in the papers so many times.’*

Patients expressed a wish for evidence-based information regarding the risks and benefits of treatment options. One patient summed up the importance to them of feeling fully informed, saying:

*‘I was not happy unless I had different opinions … we* [participant and their GP] *listed all the various questions … and wrote down their answers … When I was happy with everything then I agreed.’*

However, GPs felt the evidence base was focused on single conditions in isolation and that this made them vulnerable when calculating and conveying risks in the context of multimorbidity:

*‘The NICE guidelines focus on one problem at a time, so if we’re going to then practise outside those guidelines, some hard evidence would be helpful … so that we can communicate risks to patients who’ve got multiple problems.’*

This was a barrier to GPs enabling patients to make informed decisions to support personalised care:

*‘Guidelines don’t necessarily apply because they’re based on evidence which excludes these people … they’re excluded from the trials on which this evidence is likely to be based and we need to take the individual and virtually then tailor the consultation to their needs and their priorities.’*

#### Managing clinical uncertainty when facilitating shared decisions

GPs expressed uncertainty around managing the clinical care of patients with multimorbidity effectively, on account of a perceived lack of relevant evidence. GPs reported insecurity, and a sense that they were practising at the boundaries of evidence when managing these patients, stating that:

*‘The reason it’s difficult is because for some of them* [patients] *there’s evidence, for some of them there’s extrapolated evidence or there’s unknown evidence … and then somehow that needs to come to a complex discussion where it all gets weighed up with you facilitating that decision … The internal conflict for a GP* [is] *“Okay, that’s entirely fine. You don’t want me to refer you … that’s really woolly. I’ve got no evidence.”’*

Patients appeared aware of these challenges around clinical uncertainty and appreciated the GP’s honesty when making a shared decision. One patient recalled:

*‘The doctor said to me “You’ve got so much wrong with you I don’t know where to start”. He said, “You’ve got more wrong with you than most of my patients put together … It’s making my job very, very difficult” … The doctor was very honest with me straight at the start about everything and that’s the way to be … and you say, “Yeah, I’m going to take the chance.”’*

GPs expressed a desire for further support with managing clinical uncertainty in the context of SDM, saying that:

*‘A tool on quantifying risk and a tool on how we weigh up patient preferences with government preferences would be really, really helpful”.* They requested guidance on satisfactorily recording such information, discussing that, *“…as soon as you’re in woolly territory, you’re effectively just going...it’s just a shrug and you go, ‘Well, you can do what you like.’ And then you have to record that properly and, medico-legally, that might not stand up. You might feel a bit vulnerable.”*

#### Clinician self-efficacy for SDM

Some GPs were confident that they facilitated SDM, saying:

*‘This isn’t ground-breaking. This looks like what we probably all do anyway without being that consciously aware of it.’*

Others recognised that GPs might need to improve on SDM:

*‘I was thinking, we know that we’re pretty entrenched, and we all think we’re fantastic at this and we’re probably not.’*

*‘Just imagine, if we could all do this* [SDM] *, if every GP was trained for this, then patients would be a lot more on board with any plans that we make for them.’*

However, these GPs expressed a lack of self-efficacy for facilitating patients’ participation in the decision-making process. This appeared to be a dominant view and GPs expressed a need for further training in SDM in the context of multimorbidity, saying:

*‘… there’s some consultations where it would be really useful to be more confident in knowing what phrases to use and how to explain to a certain group of patients.’*

Some patients reported examples where GPs appeared to lack confidence to effectively involve them in a discussion of management options in the context of multimorbidity, reflecting that *‘You’re dealing with another human being who’s got her own constraints.’* They discussed examples of feeling that the GP avoided a challenging discussion by referring them on, for example:

*‘It’s usually, “Oh I’ll get you a consultant” … having a long-term problem, I think, is more difficult than going in and saying, “I’ve just got this”, and they say, “Take this.”’*

### Perceptions of time pressure and the relevance of continuity of care

Patient and GP participants identified time pressure as a barrier to effective SDM. GPs shared the opinion that:

*‘There are guidelines in terms of how you should do shared decision making, but there is no time to do it.’*

Participants reported that the process requires adequate consultation length, and/or the opportunity for successive consultations with the same GP. Patients expressed that without adequate time for a conversation, they felt less able to ‘open up’, which reduced the likelihood of a meaningful discussion about their personal priorities:

*‘I go to the doctor and I say, please give me the options that you think are going to help me and I would like to know your opinion, and then I can make an informed decision about it. But there’s never time. There’s never enough time to do that.’*

Both participant groups identified the importance of building the doctor–patient relationship and allowing a cumulative, mutual understanding to inform the decision-making process. GPs felt that:

*‘When you have continuity of care in the practice, you may begin to know your patients very well and you’re not just making a* [shared] *decision on one consultation. You’ve known them for years and you know their likes and dislikes; they know you, they know how you might treat them.’*

Patients reported finding it ‘upsetting’:

*‘*… *when you don’t get to see your own doctor … you see somebody who’s a complete stranger … I freeze.’*

## DISCUSSION

### Summary

At a time when personalised, patient-centred care is a priority in UK healthcare policy,^29^ this study reports new findings from the perspective of older patients with multimorbidity, and their GPs, regarding the challenges of SDM. A key finding was the highlighting of medicolegal vulnerability as a unifying theme for other perceived factors affecting SDM. This theme was identified independently by both patient participants and GPs. Participants discussed the challenges of applying existing clinical guidelines, clinician uncertainty and self-efficacy, and consultation duration and continuity of care.

### Strengths and limitations

Rigorous qualitative approaches were adopted in collecting and analysing data. The participant sample was heterogeneous by age, sex, practice setting, years post-qualification (GPs) and number of medical conditions (patients), with the potential for transferability of findings to a wider context.^47^ The patient sample was not ethnically diverse however was in keeping with the local demographic,^48^ and the study considered consultations with GP clinicians only. There was no minimum time required for the specified long-term conditions, which allowed for breadth of patient experience of duration and burden of illness.

The focus group facilitator was neither a doctor nor known to participants, and was thus able to act independently. Involving GP researchers provided useful insight into the consultation experience. However, they were alert to how their experiences as clinicians might influence their interpretation of the data and employed reflexivity, in this respect. The study benefitted from holding GP and patient focus groups independently, avoiding power imbalances between patients and GPs and allowing for triangulation of data. Common themes were generated across the four focus groups. Contradictory views were uncommon despite actively being sought.

### Comparison with existing literature

GPs’ concerns about medicolegal vulnerability in the context of managing multimorbidity has been previously reported.^49^^,^^50^ However, older patients’ acknowledgement of GPs’ medicolegal vulnerability has not previously been described. This study uniquely highlights how perceptions of medicolegal vulnerability underlie many barriers to SDM for older patients with multimorbidity and their GPs.

Previous studies with GPs reported constraints on personalised care driven by an expectation to follow clinical guidelines,^50^^–^53 with potentially inappropriate treatment and polypharmacy resulting.^12^^,^^28^^,^^52^^,^^52^^–^56 Although differences in healthcare setting must be acknowledged when drawing comparisons with UK general practice, a focus group study in the US explored a broader perspective by including other primary care clinicians as participants. The authors reported that, while there was variability in perceptions, some participants reported that all guidelines should be followed to ensure positive patient outcomes. Medicolegal concerns were not mentioned.^54^

Awareness of risk when sharing decisions with patients without an applicable evidence base has previously been described by GPs.^50^ The medicolegal concerns surrounding this, expressed by this study’s participants, are known to influence GPs’ behaviour towards overtreatment and potentially inappropriate referrals.^57^^–^60 Participants recognised a need for decision-support tools, previously acknowledged in the context of deprescribing for older patients,^61^ to support their management of clinical uncertainty. Guidelines for the management of multimorbidity,^28^^,^^62^^,^^63^ which recommend a personalised discussion of the pros and cons of treatments, were not well recognised by GPs in this study.

Participants recognised a need for support to communicate uncertainty comfortably and effectively.^64^^,^^65^ While advocated,^66^ there is little evidence regarding uncertainty management in primary care,^67^ or as a component of SDM.^65^ Patients’ preferences for communication of uncertainty are poorly understood.^68^ However, clinicians are known to withhold treatment options for which there is clinical uncertainty, due to concerns about patients’ reactions to ambiguous information.^69^

Many clinicians feel that they effectively facilitate SDM.^70^ Some GP participants recognised the gaps in their knowledge; however, there is a concern that others do not.^70^ In general, clinicians’ ability to facilitate SDM is low,^71^^–^73 with calls for further training.^50^^,^^74^ While educational programmes on SDM are available to clinicians, there is a lack of pragmatic guidance on how to apply the training in day-to-day general practice.^70^^,^^75^ There is currently limited evidence to guide the development of training programmes^76^ or to increase uptake of SDM.^77^

There is no apparent association between increased consultation length and improved patient satisfaction or health outcomes.^78^ However, when clinicians spend more time describing treatment options, patients are more likely to adhere to treatments and perceive greater practitioner empathy.^79^^,^^80^ Time pressures are therefore reported as a barrier to SDM in the context of multimorbidity^50^^,^^70^^,^^74^^,^^81^^–^84 and the older patients in this study reported being less able to ‘open up’ without adequate consultation duration. There is reported association between longer consultations and improved patient enablement for patients with complex needs.^78^^,^^85^

Improved continuity of care has been advocated as a facilitator of effective SDM.^50^ This aligns with the views of the older patient participants, who are recognised to particularly desire continuity with their trusted GP.^86^ Participants saw improved continuity as a solution to short consultation duration.^87^

### Implications for research and practice

To the authors’ knowledge, this study is the first to report patients’ acknowledgement of the medicolegal vulnerability of the clinician in the context of consultations for older people with multimorbidity, and their recognition of this as a barrier to SDM. Findings suggest that these perceptions influence both patient and GP behaviours. As a consequence of their awareness of the medicolegal vulnerability of the GP, patients do not appear to expect an individualised approach to clinical decision making, and opportunities for appropriate conservative management may be missed as a result. Patients’ response to an awareness of the GP’s clinical uncertainty includes their wish to feel fully informed. However, patients also appear more open to, and satisfied with, SDM when the GP is honest about their uncertainty surrounding a lack of evidence. Research is warranted to further understand how perceptions of medicolegal vulnerability may influence future interventions to facilitate SDM for this patient group in general practice.

Greater clinician awareness of guidelines that advocate the use of SDM, including those relating to the management of multimorbidity,^28^^,^^40^ appears warranted. Educational programme developers and policymakers should seek to improve dissemination and uptake of such guidelines by clinicians. Consideration of the role of QOF in helping or hindering this process would be of value. Concerns around medicolegal vulnerability and ‘fear of making mistakes’ have been linked to clinicians leaving UK clinical practice.^88^^,^^89^ Advocating an individualised, holistic approach to decision making and seeking to allay medicolegal fears faced by GPs when deviating from condition-specific guidelines may help address workforce retention. There may be a role for third-party involvement when seeking a holistic approach to care, which could be explored in future research.

While it may alleviate perceptions of medicolegal vulnerability, developing an evidence base to support all potential clinical scenarios in the context of multimorbidity is unlikely to be achievable. However, researchers could seek to provide evidence and decision-support tools for common scenarios. Both patients and clinicians should be involved in the development of guidelines of relevance to this patient group.

The present study findings suggest that having the confidence and competence to manage clinical uncertainty in a safe and effective way would help to relieve GPs’ perceptions of medicolegal vulnerability. Training should be designed to increase clinicians’ awareness that communicating uncertainty is an important component of SDM. Further research regarding patient preferences for SDM, and exploring relevant outcome measures to evaluate interventions designed to facilitate SDM, could usefully inform clinical practice. This study informed the refinement of a new intervention (VOLITION)^42^ ahead of testing its implementation and integration into practice. The refined patient component of VOLITION now informs patients to expect a tailored, individualised approach to collaborative decision making with their GP. The model of communication skills used to train GPs now includes additional training in the communication of uncertainty.

GPs need to be aware that the majority of clinicians are not already facilitating SDM effectively.^70^^,^^73^ This could be a key message in training programmes. An up-to-date systematic review of studies evaluating the effectiveness of recently developed SDM training is warranted.^90^^–^92

Policymakers and commissioning groups could consider organisational strategies to preserve adequate consultation duration and relational continuity between older patients and GPs.

Issues regarding medicolegal vulnerability underpin concerns identified by GPs and older patients with multimorbidity when considering barriers to SDM. Such issues may be addressed by targeting consulting behaviours. Improving GPs’ utilisation of multimorbidity guidelines, their communication of uncertainty, and their awareness of the need to enhance SDM for this complex and expanding patient group, is needed.
